# Interrelationships among sedentary time, sleep duration, and the metabolic syndrome in adults

**DOI:** 10.1186/1471-2458-14-666

**Published:** 2014-06-30

**Authors:** Donna Saleh, Ian Janssen

**Affiliations:** 1 Department of Public Health Sciences, Queen’s University, Kingston, ON, Canada; 2 School of Kinesiology and Health Studies, Queen’s University, Kingston ON K7L 3N6, Canada

## Abstract

**Background:**

The study objectives were to examine whether: 1) sedentary time is associated with sleep duration, 2) sedentary time predicts the metabolic syndrome (MetS) independent of sleep duration and vice versa, and 3) sedentary time and sleep duration have an interactive effect on the MetS.

**Methods:**

This cross-sectional study is based on the 2003–2006 National Health and Nutrition Examination Survey. A sample of 1371 adults (aged ≥20 years) were studied. Average daily sedentary time and sleep duration were determined via 7-day accelerometry. Screen time was determined via questionnaire. The MetS was determined using standard criteria. Analysis of variance was used to examine relationships among sedentary time and screen time with sleep duration. Logistic regression was used to examine associations between sedentary time, screen time, and sleep duration with the MetS after controlling for several confounders.

**Results:**

Sedentary time and screen time did not vary across sleep duration quartiles. Participants in the highest quartile of sedentary time were more likely to have the MetS than participants in the lowest quartile (odds ratio = 1.60, 95% CI:1.05-2.45). The odds of the MetS was higher in participants in the highest screen time tertile as compared to the lowest tertile (odds ratio = 1.67, 95% confidence interval:1.13-2.48). Sleep duration was not independently related to the MetS. There were no significant sedentary time X sleep duration interactions on the MetS.

**Conclusion:**

Highly sedentary individuals and individuals with a high screen time are more likely to have the MetS.

## Background

Research on the health benefits of movement has traditionally focused on a lack of moderate-to-vigorous physical activity (MVPA) [1]. Recently, homage has also been given to excessive sedentary behavior [1,2] and insufficient sleep [3,4]. Sedentary behavior is defined as time spent sitting or lying during waking hours [5] while insufficient sleep is defined as sleeping less than 7 hours/day [6].

Time spent being sedentary may be connected to sleep duration. Large cross-sectional studies of children have shown that excessive TV watching and computer use are associated with short sleep duration [7-9]. An experimental study of 18 overweight adults showed that the proportion of waking hours spent sedentary increased from 83% to 87% after sleep was restricted by 3 hours [10]. Participants in that study reported feeling more weary and had less vigor after sleep restriction. Since few studies have addressed the association between sedentary time and sleep duration in adults, additional research is warranted.

Sedentary time and sleep duration have both been linked to the metabolic syndrome (MetS), a clustering of risk factors for cardiovascular disease and type 2 diabetes, and other cardiometabolic risk factors [1-4,11-16]. We are only aware of one study that has simultaneously examined sedentary time and sleep duration [14]. That study reported that reallocating 30 minutes/day from sedentary time to sleep was associated with about a 2% difference in insulin sensitivity. However, that study used a self-reported measure of sleep duration and validation studies indicate that people have difficulties assessing their own sleep and report biased estimates [17,18]. Furthermore, that study did not determine whether the association between sedentary time and cardiometabolic health is moderated by sleep duration and/or whether the association between sleep duration and cardiometabolic health is moderated by sedentary time.

This study had three objectives. First, we examined the association between sedentary time and sleep duration in a large sample of adults. Second, we examined whether sedentary time predicted the MetS independent of sleep duration and vice versa. Third, we examined whether sedentary time and sleep duration had an interactive effect on the MetS.

## Methods

### Overview of study design and measures

Study data are from the 2003–2004 and 2005–2006 cycles of the U.S. National Health and Nutrition Examination Survey (NHANES). NHANES is a nationally representative cross-sectional study that assesses the health of adults and children. It combines interviews, physical examinations, and laboratory tests that take place in a home interview and mobile examination center visit. NHANES uses a complex, multistage probability sampling design to select participants. Sampling weights were applied in the analysis to reflect the unequal probability of participation among certain demographic groups.

NHANES was approved by the National Center for Health Statistics Research Ethics Review Board and participants provided informed consent. Ethics approval for the secondary analysis conducted for this study was obtained from the Queen’s University Health Sciences Research Ethics Board.

### Participants

The present study was limited to non-pregnant adult (aged ≥20 years) participants without chronic disease (cancer, diabetes, coronary heart disease, stroke, chronic bronchitis and emphysema) who comprised the morning fasting subsample. Of the 3373 participants who met these eligibility criteria, 191 were excluded because they were missing one or more components of the MetS, 1462 were excluded because they did not have valid accelerometer data for the sedentary time and/or sleep duration measures, and 349 were excluded because they were missing one or more of the covariates. Thus, the final sample consisted of 1371 participants. Although many participants were lost because they failed to meet the eligibility criteria, only 6% of participants who were in the morning subsample were lost because they did not have sufficient MetS information and it was therefore acceptable to continue the analysis. Participants who were excluded from this study were similar in age (51 vs. 49 years) and ethnicity (52% vs. 54% non-Hispanic white) to those who were included. However, more females than males were excluded (56% vs. 50% male), which was largely explained by the exclusion of 330 pregnant women.

### Sedentary time

Time spent being sedentary was measured using the uniaxial Actigraph AM-7164 accelerometer. Accelerometers are small electronic devices which assess, minute-by-minute data on the volume, intensity, duration and frequency of most movement. Accelerometers provide a reliable and valid measure of sedentary time. When assessed against the activePAL, a triaxial inclinometer that differentiates between sitting and standing, correlations of sedentary time are high (r = 0.76) [19].

Participants were given the accelerometer at the mobile exam center visit and instructed to wear it for the following 7 days over their right hip using the elastic belt provided, and to remove the monitor before going to bed and during showers, bathing, and swimming. The accelerometers provided 10,080 consecutive minute-by-minute movement data points (e.g., one data point for each minute of the week). After the 7 day measurement period accelerometers were returned to the NHANES investigators by mail.

The first stage of accelerometer data cleaning was conducted by NHANES investigators who removed outliers or biologically implausible values. The remaining data cleaning and reduction was completed by the authors using established criteria [20,21]. Initially, periods of non-wear time were removed. Non-wear time was defined as an interval of at least 60 consecutive minutes of 0 activity intensity counts, with allowance for 1–2 min of counts above 0 [20,21]. In the next step, days that were invalid were removed. A valid day was defined as a day with at least 10 hours of wear time [20,21]. The next step was to remove participants with an insufficient number of valid days. Only participants with at least 4 valid days were included in the analysis [20,21].

Next, each minute of accelerometer data was defined as being sedentary or of a light or moderate-to-vigorous intensity using established cut-points. A given minute was considered sedentary if the accelerometer count value did not exceed the 100 counts per minute threshold [12,22]. Counts ranging from 100–2020 were classified as light intensity activity and counts of 2020 or higher were considered MVPA [20]. Total sedentary time and time spent in light intensity physical activity and MVPA were calculated for each valid day by summing the number of minutes, and then averaged across all valid days. Wear time was calculated by subtracting non-wear time from 24 hours. The proportion of total wear time that was sedentary was then determined. The proportion of total wear time that was sedentary was divided into quartiles as following: Q1 = 21.0-48.1%, Q2 = 48.2-56.5%, Q3 = 56.5-63.9%, Q4 = 64.0-90.7%.

### Screen time

The second exposure variable measured total screen time (including TV, video, and computer use) using the interview questions, “Over the past 30 days, on average how many hours per day did you sit and watch TV or videos?” and “Over the past 30 days, on average how many hours per day did you use a computer or play computer games?” There were 7 response options for each question: 0 hours, less than 1 hour, 1 hour, 2 hours, 3 hours, 4 hours, and 5 or more hours. TV and computer time were summed to create an overall screen time score. Screen time was then divided into tertiles. The range of screen time (hours/day) for each tertile is as follows: T1 = 0–1, T2 = 2, T3 = 3-6. Questionnaires measuring TV time, such as the NHANES questionnaire, are moderately correlated with TV time measured by a detailed log (r = 0.47) [23].

### Sleep duration

While population-based studies have typically used self-report questionnaires to gather sleep duration data, self-report data are poorly correlated with criterion measures and are systematically biased [17,18]. Therefore, in this study sleep duration was estimated using an objective proxy measure. Using data gathered from accelerometry, the longest period of non-wear time in the 24-hour period between 12:00 noon on two valid days was used as a proxy for sleep duration. The same criteria as explained above under sedentary time were used to define non-wear time periods and valid days. Only those who had ≥2 valid sleep night periods were included in the analysis. The criteria for having ≥2 valid sleep nights was determined by examining the correlation between the sleep duration proxy measures from participants who had complete (6 nights) sleep data. These analyses revealed the flowing correlations with the average sleep duration obtained over 6 nights: r = 0.77 for one randomly chosen night, r = 0.84 for two randomly chosen nights, r = 0.93 for three randomly chosen nights, r = 0.95 for four randomly chosen nights, and r = 0.98 for 5 randomly chosen nights. Because the correlation for 2 nights (r = 0.84) is very strong, and because there was a large drop in sample size with ≥2 nights of valid data vs. ≥3 nights of valid data (n = 1371 vs. n = 984), ≥2 nights was chosen as the criteria.

The average proxy sleep duration was calculated by averaging the sleep duration across the number of nights with valid data and then divided into quartiles as follows: Q1 = 3.0-7.2 hours/night, Q2 = 7.2-8.6 hours/night, Q3 = 8.6-9.7 hours/night, Q4 = 9.7-11.8 hours/night. Extreme sleep duration observations (below 2^nd^ and above 98^th^ percentile) were deleted since they were thought to inaccurately represent sleep duration as because they were outside the physiological range of sleep duration times. These values could have resulted from participants removing their accelerometer before bed or a temporary error with the accelerometer device that can cause high count values to sporadically occur [24].

### Metabolic syndrome

Participants were classified as having the MetS using standard criteria [25] based on having three or more of the following five risk factors: high waist circumference (≥94 cm in men, ≥80 cm in women), high triglycerides (≥150 mg/dL), low HDL-cholesterol (<40 mg/dL in men, <50 mg/dL in women), high blood pressure (systolic ≥130 mmHg or diastolic ≥85 mmHg or medication use), and high blood glucose (≥100 mg/dL or presences of diabetes or medication use).

All MetS component data were taken by trained technicians. Waist circumference was obtained to the closest 0.1 cm using a flexible tape at the level of the iliac crest. Prior to measuring blood pressure, participants rested quietly in a seated position for 5 minutes. Then, four blood pressure readings were obtained using a manual mercury sphygmomanometer. Blood pressure measurements were averaged for each participant. Blood was drawn from an antecubital vein of the left arm following an overnight fast. HDL-cholesterol was measured using the direct HDL immunoassay method [26]. Cholesterol and triglycerides were measured enzymatically in a series of coupled reactions hydrolyzing cholesterol ester and triglyceride to cholesterol and glycerol, respectively [27]. Fasting plasma glucose was determined using a hexokinase enzymatic method [28]. The presence of physician diagnosed diabetes (other than gestational diabetes) and medication use for diabetes and hypertension were assessed in the interview.

### Confounders

Age, sex, ethnicity (non-Hispanic white, non-Hispanic black, Hispanic, other), education level (less than high school, high school graduate, college graduate), socioeconomic status (SES), smoking status (never, former or current), alcohol consumption, caffeine consumption, and physical activity were considered as confounders. SES was assessed using the poverty-to-income ratio, which is a ratio between family income and the poverty threshold [29]. Caffeine consumption was assessed using a 24-hour food recall and those who consumed more than 250 mg/day of caffeine were considered high caffeine users [30]. Females who consumed more than 7 alcoholic drinks/week and males who consumed more than 14 alcoholic drinks/week were considered excessive alcohol users [31]. Finally, MVPA was assessed by accelerometry as previously described [20]. Mean duration of MVPA per day accumulated in bouts of at least 10 minutes was calculated for all participants and three groups were created: no bouts of MVPA, some MVPA (up to 75 minutes/week), and at least 75 minutes/week of MVPA. These cut points are based on half of the minimum physical activity in the public health guidelines [32].

### Statistical analysis

All data were analyzed using the SAS 9.2 Software (SAS Inc., Cary, NC) and took into consideration the complex survey design and sample weights. Descriptive statistics were used to determine baseline characteristics of the study population. Relations between sedentary time and sleep duration variables were determined using Pearson correlations. ANOVA were used to explore the relationship between sleep duration with the proportion of total wear time that was sedentary and screen time. Multiple logistic regression was performed to examine the relationship between the sedentary time and sleep duration variables with the MetS and its individual components. Initially, univariate models were used to describe the associations between the sedentary time and sleep duration variables with the MetS. This was followed by two multivariate models. The first multivariate model included screen time, sleep duration, and the covariates. Total sedentary time replaced screen time in the second multivariate model. To adjust for potential confounders in the multivariate models, the backward deletion according to change in estimate criteria approach was used [33]. Therefore, after starting with a full model of all confounders, potential confounders that did not change the risk estimate for the MetS for at least one of the key exposures (sedentary time or sleep duration) by more than 5% were removed in a stepwise fashion. Finally, sedentary time (or screen time) X sleep duration interaction terms were added to the models to assess whether sleep had a moderating effect on the sedentary time-MetS relationship.

## Results

### Descriptive characteristics

Participant characteristics are in Table 1. Of the 1371 participants, approximately 56% were male and the mean age was 49 years. Overall, 56% of total accelerometry wear time was spent sedentary. The proxy sleep duration was 8.3 hours per night. Participants watched 2.2 hours/day of TV and used the computer for 44 minutes/day, for a total of 2.3 hours/day of screen time.

**Table 1:** Characteristics of the study sample

**Characteristic**	**N = 1371**	**Prevalence (%)**	**Mean (SD)**
***Demographic Factors***			
Sex, % male	770	56.2	-
Age (years)			
20-39	451	32.9	-
40-59	523	38.2	
60+	397	29.0	
Ethnicity			-
Non-Hispanic white	738	53.8	
Non-Hispanic black	245	17.9	
Hispanic	334	24.4	
Other	54	3.9	
Education			-
< High school	313	22.8	
High school graduate	742	54.1	
College graduate	316	23.1	
Poverty level, % below poverty	153	11.2	-
***Behavioral Factors***			
Caffeine consumption, % ≥250 mg/day	284	20.7	-
Smoking status			-
Never	669	48.8	
Former	404	29.5	
Current	298	21.7	
Alcohol consumption (drinks/week)			-
None	312	22.8	
Some	1040	75.9	
Excessive	19	1.4	
Sedentary Behavior	-	-	
Screen time (hours/day)			2.3 (1.0)
% of total wear time that is sedentary			56.2 (11.3)
Sleep duration proxy (hours/day)			8.3 (1.9)
Moderate-to-vigorous physical activity (min/day)	-	-	7.0 (12.5)
***Metabolic Syndrome Components***			
Metabolic syndrome, % yes	512	37.4	-
Waist circumference (cm), % high	987	72.0	97 (14)
HDL-cholesterol (mg/dL), % low	418	30.5	55 (16)
Triglycerides (mg/dL), % high	416	30.3	140 (113)
Glucose (mg/dL), % high	521	38.0	99 (17)
Systolic blood pressure (mmHg), % high	518	37.8	124 (17)
Diastolic blood pressure (mmHg), % high	214	15.6	71 (11)

### Relationships between sedentary time and sleep duration variables

Total sedentary time and screen time were weakly correlated (r = 0.18). Sedentary time and screen time were poorly correlated to sleep duration (r = 0.04 and r = 0.004, respectively). As shown in Table 2, sedentary time and screen time means did not vary across the sleep duration quartiles (p = 0.08 and p = 0.87, respectively).

**Table 2:** Means and adjusted means* of the sedentary behavior variables according to sleep duration

**Sleep duration quartile**	**Screen time (hours/day)**		**% of total wear time that is sedentary**	
	**Mean (SD)**	Adjusted mean* (SD)	**Mean (SD)**	Adjusted mean *(SD)
Q1 (shortest sleep)	2.35 (1.05)	2.40 (2.22)	56.05 (11.01)	57.72 (22.77)
Q2	2.30 (0.92)	2.41 (1.98)	58.13 (10.76)	57.36 (23.51)
Q3	2.30 (0.97)	2.47 (2.09)	57.59 (10.94)	56.57 (23.14)
Q4 (longest sleep)	2.35 (0.90)	2.50 (1.94)	57.91 (10.65)	56.23 (23.51)
p-value	0.87	0.87	0.13	0.08

*Adjusted for all sex, age, ethnicity, education, poverty level, caffeine consumption, alcohol consumption and MVPA.

### Relationship between sedentary time and sleep duration with the MetS

Of those participants who accrued the most screen time, 44% had the MetS. Conversely, only 29% in the lowest screen time tertile had the MetS. Of the most sedentary participants, 46% had the MetS; only 32% in the lowest sedentary time quartile had the MetS. There was no trend in the prevalence of the MetS across sleep duration groups (Table 3).

**Table 3:** Prevalence and odds ratio (95% confidence interval) for the metabolic syndrome according to sedentary time and sleep duration

**Exposure or *c*** **onfounder**	**Prevalence, %**	**Univariate models**	Multivariate model 1*	Multivariate model 2**
***Exposures***				
Screen time				
T1 (least screen time)	29.3	1.00 (ref)	1.00 (ref)	
T2	35.3	1.32 (0.96-1.81)	1.18 (0.81-1.72)	
T3 (most screen time)	44.1	1.91 (1.38-2.64)	1.67 (1.13-2.48)	
Sedentary time				
Q1 (least sedentary)	32.4	1.00 (ref)		1.00 (ref)
Q2	34.1	1.18 (0.80-1.76)		1.00 (0.66-1.51)
Q3	37.0	1.44 (0.98-2.1)		1.25 (0.83-1.89)
Q4 (most sedentary)	45.9	2.10 (1.44-3.06)		1.60 (1.05-2.45)
Sleep duration				
Q1 (shortest sleep)	34.1	0.86 (0.60-1.23)	0.90 (0.62-1.32)	0.91 (0.62-1.33)
Q2	37.4	1.00 (ref)	1.00 (ref)	1.00 (ref)
Q3	35.6	0.93 (0.65-1.33)	0.89 (0.61-1.30)	0.89 (0.61-1.29)
Q4 (longest sleep)	39.8	1.12 (0.78-1.60)	0.97 (0.67-1.41)	0.95 (0.66-1.39)
***Relevant Confounders*** *†*				
Age				
20-39 y	23.3	1.00 (ref)	1.00 (ref)	1.00 (ref)
40-59 y	37.1	2.24 (1.60-3.13)	2.29 (1.63-3.22)	2.23 (1.58-3.13)
60+ y	53.7	4.00 (2.82-5.67)	4.00 (2.81-5.71)	3.13 (2.16-4.54)
Ethnicity				
Non-Hispanic white	39.2	1.00 (ref)	1.00 (ref)	
Non-Hispanic black	29.8	0.60 (0.42-0.87)	0.60 (0.41-0.88)	
Hispanic	38.0	0.96 (0.70-1.31)	1.10 (0.79-1.54)	
Other	42.6	1.45 (0.77-2.71)	1.55 (0.80-3.00)	
Education				
Less than high school	44.4	1.00 (ref)		1.00 (ref)
High school graduate	37.3	0.78 (0.56-1.07)		0.86 (0.61-1.21)
College graduate	30.4	0.61 (0.42-0.89)		0.69 (0.45-1.03)
Moderate-to-vigorous physical activity				
None	44.3	1.00 (ref)		1.00 (ref)
Some	34.7	0.54 (0.39-0.74)		0.70 (0.49-0.98)
Moderate	25.8	0.46 (0.33-0.64)		0.58 (0.41-0.81)
***Remaining Confounders***				
Sex				
Male	40.5	1.00 (ref)		
Female	33.3	0.75 (0.58-0.97)		
Poverty level				
Below	43.8	1.36 (0.91-2.03)		
Above	36.5	1.00 (ref)		
Caffeine				
<250 mg/day	36.7	1.00 (ref)		
> = 250 mg/day	39.8	1.18 (0.87-1.61)		
Smoking Status				
Never	35.1	1.00 (ref)		
Former	42.8	1.45 (1.08-1.95)		
Current	34.9	0.93 (0.67-1.30)		
Alcohol consumption				
None	45.2	1.52 (1.13-2.05)		
Some	35.1	1.00 (ref)		
Excessive	31.6	0.57 (0.16-2.08)		

*Multivariate model 1 includes screen time and sleep duration as the main exposures.

**Multivariate model 2 includes sedentary time and sleep duration as the main exposures.

†The backward deletion according the change in estimate approach was used to identify relevant confounders. Confounders that changed the odds ratio for the main exposures by >5% were kept in the model and those that did not were removed.

After adjusting for relevant confounders (age, education level, MVPA) and sleep duration, the relative odds of the MetS was higher in participants in the highest sedentary time quartile by comparison to participants in the lowest quartile (odds ratio (OR) = 1.60, 95% confidence interval (CI): 1.05-2.45). A positive relationship was also observed with screen time such that the odds ratio of the MetS was higher in participants in the highest screen time tertile as compared to the lowest tertile (OR = 1.67, 95% CI:1.13-2.48). Sleep duration was not related to the MetS in the bivariate or multivariate logistic regression models. There were no significant sedentary time X sleep duration or screen time X sleep duration interactions in any of the models.

### Relationship between sedentary time and sleep duration with the MetS components

As shown in Table 4, sedentary time and screen time were significantly associated with a high waist circumference, high triglycerides, and a low HLD-cholesterol (sedentary time only). The associations between sleep duration and the MetS components were weak and non-significant. There were no significant sedentary time X sleep duration or screen time X sleep duration interactions in any of the models.

**Table 4:** Odds ratio (95% confidence interval) for the metabolic syndrome components according to sleep duration and sedentary time

**Exposure**	High triglycerides^*^	High plasma glucose^**^	**High waist circumference**†	**High blood pressure**^‡^	**Low HDL-cholesterol**^*¶*^
Sleep duration					
Q1 (shortest sleep)	0.90 (0.61-1.33)	1.08 (0.74-1.59)	1.25 (0.85-1.84)	1.12 (0.76-1.65)	1.20 (0.78-1.85)
Q2	1.00 (ref)	1.00 (ref)	1.00 (ref)	1.00 (ref)	1.00 (ref)
Q3	1.26 (0.86-1.84)	1.03 (0.70-1.50)	1.07 (0.72-1.57)	0.89 (0.60-1.31)	0.89 (0.59-1.34)
Q4 (longest sleep)	1.13 (0.77-1.66)	0.96 (0.66-1.41)	1.26 (0.84-1.87)	1.15 (0.78-1.70)	1.23 (0.80-1.89)
Screen time					
T1 (least screen time)	1.00 (ref)	1.00 (ref)	1.00 (ref)	1.00 (ref)	1.00 (ref)
T2	1.56 (1.03-2.37)	0.94 (0.64-1.38)	1.38 (0.93-2.04)	1.02 (0.68-1.52)	0.79 (0.50-1.23)
T3 (most screen time)	1.71 (1.11-2.63)	0.76 (0.51-1.13)	1.53 (1.09-2.32)	1.43 (0.94-2.17)	0.65 (0.41-1.03)
Sedentary time					
Q1 (least sedentary)	1.00 (ref)	1.00 (ref)	1.00 (ref)	1.00 (ref)	1.00 (ref)
Q2	1.23 (0.78-1.93)	1.46 (0.97-2.22)	1.48 (0.97-2.26)	0.87 (0.57-1.34)	0.64 (0.40-1.04)
Q3	1.85 (1.18-2.88)	1.07 (0.71-1.61)	1.83 (1.19-2.81)	1.15 (0.75-1.77)	0.90 (0.55-1.48)
Q4 (most sedentary)	1.81 (1.15-2.85)	0.86 (0.57-1.31)	1.81 (1.16-2.83)	0.94 (0.59-1.47)	0.56 (0.35-0.92)

^*^Alcohol consumption was the only significant confounder.

^**^No significant confounders.

†Significant confounders include sex, ethnicity, and smoking status.

^‡^Age was the only significant confounder.

^¶^Smoking was the only significant confounder.

## Discussion

This study examined associations between sedentary time, sleep duration, and the MetS in adults. Sedentary time and sleep duration were not correlated. There were moderate associations between sedentary time and screen time with the MetS; however, sleep duration was not associated with the MetS. Sedentary time and sleep duration did not have an interactive effect on the MetS.

Results from this study indicate that the average adult spends over half of their waking hours being sedentary. Adults who spend between 65-90% of their day sedentary were more likely (OR = 1.60, 95% CI:1.05-2.45) to have the MetS than those who spent less than 48% of their day sedentary. This result is consistent with a previous study that used the NHANES dataset [12], and with several other cross-sectional studies that report moderate associations between sedentary time and cardiometabolic risk [15]. Additionally, the strongest associations with sedentary time were observed for waist circumference and triglycerides, which is consistent with previous evidence [12]. We extended existing knowledge by demonstrating that the relationship between sedentary time and the MetS was not moderated by sleep duration. In other words, these relationships were consistent in short, medium, and long sleepers.

Adults who spent more than 3 hours per day in front of TV and computer screens were at increased odds (OR = 1.67, 95% CI:1.13-2.48) for having the MetS compared to those who spent less than 1 hour per day, which is also consistent with previous literature [13,16]. These findings are important because they provide context to the type of sedentary behavior that is likely to confer a health risk. Futures studies would benefit from examining other types of sedentary behavior outside of screen time since little is known about the health impact of non-screen based sedentary behavior and because different sedentary behaviors may require distinct interventions.

Sleep duration was unrelated to sedentary time and screen time in this large and diverse sample of adults. This is contrary to observational findings reported in children and youth [7-9] and experimental studies from a small (n = 18) sample of adults [10]. It is unclear what accounts for these discrepant findings.

Although sleep duration was not associated with the MetS in this study, there was a borderline positive association with sleep duration and waist circumference, which is consistent with recent evidence indicating that short sleep is associated with abdominal adiposity [34]. Although previous studies examining this relationship found stronger associations with sleep duration and obesity, most of them have relied on self-report methods to capture sleep duration. If the misclassification associated with self-reported sleep duration measures is differential, that could explain the different results. Future studies should considering control for obstructive sleep apnea, which may be a significant confounder, and should explore relationships with sleep onset timing rather than sleep duration, since timing is a factor that is related to unhealthy eating behaviors [35], circadian rhythm disruption [36], and melatonin suppression [36].

Although it is still unclear how much sedentary time leads to an increased health risk, this study has shown a monotonic relationship, with more sedentary time leading to an increased odds of the MetS. Therefore, time spent being sedentary is significant, even if the only plausible explanation is that it displaces time spent in light-intensity physical activity, leading to a reduction in overall energy expenditure [37]. Even substituting 2 hours/day of sedentary time (1.5 METS) with light-intensity activity (2.5 METs) would be the equivalent of a 30 minute brisk walk [37]. A recent randomized control study showed the significant impact of making this simple substitution. Adults who participated in a TV commercial stepping program (replaced sitting screen-time with light-intensity physical activity) had significant decreases in their percent body fat and waist circumference over a 6 month period [38]. Altogether, this evidence has important implications for future public health initiatives and interventions. A next step would be to identify the key modifiable factors lead to excessive sedentary time, as the determinants of sedentary time have not been studied extensively [39].

An important strength of this study is that sedentary time and sleep duration were measured objectively. Additionally, we used waist circumference, which has been shown to explain obesity-related health risk to a greater extent than BMI [40]. Our study has important limitations. Firstly, temporal associations could not be determined due to the cross-sectional nature of this study. Also, measurement bias may have been present because of the use of self-reported data for many of the confounders and screen time. We were not able to control for or eliminate all potential confounders, most notably the presence of sleep apnea. The sleep duration measure, while objective, was still a proxy measure and it is likely that sleep duration was overestimated with this proxy measure. Finally, the accelerometers used in NHANES did not accurately capture all activities (eg, swimming, cycling, load-bearing activities) and there is a lack of consensus in the literature around the optimal accelerometer data reduction and cleaning procedures.

## Conclusion

Time spent sedentary is related to cardiometabolic risk, independent of sleep duration, although prospective evidence is needed to confirm the direction of the relationship. Additionally, more prospective research using objective measures of sleep duration, timing, and quality are needed to explore the relationship between sleep with sedentary time and cardiometabolic risk.

## Abbreviations

CI: Confidence interval; HDL: High density lipoprotein; NHANES: National health and nutrition examination survey; MetS: Metabolic syndrome; MVPA: Moderate-to-vigorous physical activity; OR: Odds ratio.

## Competing interests

The authors declare that they have no competing interests.

## Authors’ contributions

This is the work of DS under the supervision of IJ. The research question was a collaborative effort between the two authors. DS performed data cleaning and reduction of the NHANES databases, developed the SAS program to clean the accelerometer data to derive the objective sedentary time and sleep duration variables, performed all statistical analyses, interpreted the results, and wrote the original draft of the manuscript. IJ provided guidance on all aspects of the work and performed extensive revisions of the written work. Both authors read and approved the final manuscript.

## Pre-publication history

The pre-publication history for this paper can be accessed here:

http://www.biomedcentral.com/1471-2458/14/666/prepub
